# Brief Report: A Randomized Control Trial Assessing the Influence of a Telephone-based Intervention on Readmissions for Patients with Severe Mental Illness in a Developing Country

**DOI:** 10.1007/s10597-016-0069-4

**Published:** 2016-11-30

**Authors:** U. A. Botha, L. Koen, M. Mazinu, E. Jordaan, D. J. H. Niehaus

**Affiliations:** 1 Department of Psychiatry, Faculty of Medicine and Health Sciences, University of Stellenbosch, Tygerberg, PO Box 241, Cape Town, 8000 South Africa; 2 Biostatistics Unit, Medical Research Council, Parow, South Africa; 3 Statistics and Population Studies, University of the Western Cape, Cape Town, South Africa

**Keywords:** Post-discharge initiative, Telephone-based facilitation, Readmission, Days in hospital, Dual diagnosis patients

## Abstract

Whilst comprehensive post-discharge interventions have been successful in reducing readmissions in our setting, they are possibly not sustainable due to limited resources. We assessed the impact of a more cost-effective telephone-based intervention on readmissions in a developing country over 12 months. 100 patients with severe mental illness were randomized to facilitated care or treatment as usual. All were interviewed prior to discharge and after 12 months. Facilitated care consisted of structured telephonic interviews and motivational support to patients and families. At 12 months no significant differences in either readmissions (p = 0.10) or days in hospital (p = 0.44) could be demonstrated. Substance use was high (64%), particularly methamphetamine (44%) in both groups. The intervention did not have any impact on inpatient usage in our setting. Though this study was limited by its small sample size, the results indicated that affordable post-discharge services may not be comprehensive enough to reduce readmission rates and would have to be tailored to the distinct population of dual diagnosis patients identified in this study.

## Introduction

The last two decades have seen a significant increase in community-based interventions for patients with severe mental illness (Dixon 2000; Smith and Newton 2007; Marshall and Lockwood 2000; Karow et al. 2012, Boden et al. 2010; Vigod et al. 2013; Marshall et al. 2011). One of the driving forces behind this research impetus has undoubtedly been the worldwide pressure on inpatient beds, caused by the reduction of acute psychiatric beds as part of the global drive towards deinstitutionalization. In South Africa, these repercussions are experienced the same way as in many other countries in the world, but are additionally influenced by factors unique to this particular setting (Lazarus 2005; Botha et al. 2008). The public health sector in South Africa is hampered by limited community-based resources, high patient volumes and limited specialist care at community level. The majority of service users come from challenging social environments, were substance use and unemployment rates are high.

The post-discharge period is associated with high drop-out figures, with recent studies showing less than 50% of patients attend their first scheduled outpatient appointment and significant delays between discharge and outpatient follow-up (Boyer et al. 2000; Klinkenberg and Calsyn 1996). The first month after discharge is often most critical as readmissions peak during this period (Naji et al. 1999). These findings highlight the importance of post-discharge interventions that ensure smooth transition to outpatient care. Interventions employed during this period are very diverse, ranging from mere pre-discharge, once-off interventions to well-defined programs utilizing care-coordinators who facilitate care over extended periods of time (Vigod et al. 2013; Steffen et al. 2009; Nurjannah et al. 2014; Dixon et al. 2009). In a recent review of 11 studies, Steffen et al. concluded that discharge interventions were effective in reducing readmissions and improving adherence (Steffen et al. 2009).

Readmissions rates are influenced by a wide range of factors and may vary significantly depending on how psychiatric services are structured as well as the specific socio-demographic factors influencing in-patient usage in a particular setting (Loch 2012; Zhou et al. 2014; Barekatain et al. 2014). Though readmission rates are a popular outcome measure, they do not accurately reflect overall inpatient usage, since readmissions alone do not reflect length of stay (Puschner et al. 2011). To better reflect the nature of inpatient use many authors combine readmissions with cumulative days in hospital (DIH) over a given period of time (Lucas et al. 2001).

The Transitional Discharge Model focuses specifically on reducing readmissions in the first 90 days after discharge by providing a range of inputs during the pre-discharge and post-discharge period. In a recent systematic review, Vigod et al. commented that high rates of early readmissions may be an indication of the quality of in-patient care or inadequate continuity of care on discharge. The review assessed the effect of transitional care and identified specific components that were associated with a lower readmission rate. These components include psychoeducation (pre- and post-discharge), home-visits, phone-call reminders, making use of a transitional manager and communication with primary care providers. The authors concluded that a number of the transitional care components could easily be incorporated into a cost-effective intervention and urged the need for further exploration of these models (Vigod et al. 2013). The potential for cost-effective modification of the model makes it attractive to developing countries, where resources are often limited.

The recent National Mental Health Summit held in South Africa in April 2012 served as an excellent stage to reflect on the status of mental health service delivery in South Africa. The summit concluded with a policy commitment which amongst other issues, called for more comprehensive continuity of care between in-patient services and primary care providers (PCPs) in an attempt to reduce “revolving door” patients. PCPs are in most instances the principal providers of outpatient mental health care and include mental health nurses. In addition to this, there was also a call for an increase in research assessing mental health services in local settings in an attempt to instruct development of service with evidence-based arguments (Lund et al. 2012).

Also in South Africa, our group reported on a modified Assertive Community Treatment (ACT) program which produced significant reduction in readmission rates in a group of high-frequency users compared to a control group (Botha et al. 2010). A 3 year follow-up of this group reported sustained reduction in days in hospital and lower readmission rates (Botha et al. 2014). Although the intervention was modified to allow for larger case-loads and less frequent visits, it remains a comparatively costly, specialized service which is only available to a relatively small part of the population. This type of service, despite its success in reducing admissions, may not be justifiable in a developing world where resources are limited and standard care practice has so much room for improvement. Consequently, we piloted a post-discharge service which would offer less comprehensive input, could be accessible to more patients and remain affordable. The service would attempt to enhance use of existing standard care services by incorporating some care components that have been identified in the literature to be effective in reducing readmissions.

## Aim

*Primary objective* The purpose of the study was to assess the effect of a post-discharge, telephone-based intervention on readmissions and DIH in patients with severe mental illness over a 1 year period.

*Secondary objective* In addition to the main objective, we were also explored the effect of the intervention on illness severity.

### Ethical Considerations

This study was approved by the Committee for Human Research of the University of Stellenbosch (N09/10/263) and was conducted in accordance with the International Committee for Harmonisation (ICH) Good Clinical Practice (GCP) guidelines and SA GCP as well as the Declaration of Helsinki (2000). All participation was on a completely voluntary basis and patients were able to withdraw their permission at any time. The study was registered on the National Clinical Trial Register. (SANCTR no 3548).

## Methods

This was a randomized, non-blinded clinical trial conducted at Stikland Hospital in Cape Town, South Africa. Participants were randomized using block randomization, to one of two groups (a) Facilitated Care Group (FCG) and (b) Treatment as Usual Group (TUG). The allocation sequence was monitored by the principal investigator (UB). The study continued until the last included participant reached the end of the 12-month follow-up period.

*Inclusion criteria* Participants (male and female) were between the ages of 18 and 59 (inclusive), had an established diagnosis of schizophrenia, schizo-affective disorder or bipolar disorder and were able to give written, informed consent. The inclusion criteria was designed to represent the bulk of psychiatric diagnoses accounting for the pressure on the acute inpatient units.

*Exclusion criteria* Patients with moderate to severe mental retardation, unstable co-morbid medical illness, receiving other assertive interventions, those unable to provide reliable phone number on discharge and patients living more than 30 km outside the Metro Catchment Area of Stikland Hospital, were excluded. Exclusion criteria were intended to avoid biasing outcomes of the intervention.

### Study Procedure

After consent was obtained, participants (FCG + TUG) were interviewed by a member of the study team in the week prior to discharge with a semi-structured interview to collect data on demographics, medical and psychiatric history and treatment history. Substance use questionnaires and Clinical Global Impression (CGI) Scales were completed.

### Intervention Procedure

The intervention consisted of telephonic facilitation of the existing standard care service. Participants allocated to the FCG were each assigned a care facilitator (CF). CF’s were members of the local ACT team and experienced in community-based follow-up of patients with chronic mental disorders. All team members were briefly trained in providing a concise, semi-structured telephonic intervention. Participants were seen by CF’s prior to discharge and received monthly phone calls prior to clinic appointments. A single emergency home visit was allowed to re-engage participants who had lost contact. CF’s also phoned mental health care providers to confirm attendance and provide feedback.

Participants in the FCG received the intervention for 12 months. On conclusion of the study, participants in the FCG who were thought to require more comprehensive follow-up, were referred to the local ACT team. The remainder of FCG participants were referred back to standard care services.

### Standard Care

Participants allocated to TUG received the standard community mental health care as provided in their local community. This includes a wide range community-based follow-up at local mental health clinic level, provided by community mental health nurses. There is no set standard for frequency of contacts and care is generally tailored according to the service pressure and resources available.

During the exit (12-month) interview, data was collected on readmissions and treatment history of the last 12 months and CGI’s were completed. Admission data was obtained from the provincial electronic system recording all patient contacts and admissions. Participants in the FCG also completed a brief questionnaire on how the intervention was experienced and CFs were required to comment on participants’ engagement and whether they were difficult to get hold of.

### Statistical Analysis

The purpose of the study was to assess the effect of the telephone-based intervention on readmissions. All data was entered into a single database. Data was analysed and processed in consultation with a statistician. The demographic data was summarised as counts (n), frequencies and percentages (%) for categorical variables, as well as means, medians and interquartile ranges (IQR) for numerical variables. For the categorical independent variables, the Fisher’s exact test was used to assess differences in demographic and substance use patterns between the two groups (FCG and TUG). This comparison was done to check that the randomization resulted in balanced groups. The DIH and number of admissions were the primary outcomes. Histograms of the distributions for the pre- and post-outcomes were included to show the non-normality of these variables. The CGI was the secondary outcome. Since this is a randomized-controlled trial, the primary analysis is a comparison of the outcomes for the FCG and TUG groups at post-intervention. However, the pre-intervention outcomes are first checked for differences in group outcomes. If differences are found, these would be adjusted for in the post-intervention comparison. The DIH, number of admissions and CGI outcomes pre- and post- were skewed and not normal. For comparison of FCG and TUG groups, the number of admissions and CGI outcomes pre- and post- were changed to an ordinal scale (three categories) to accommodate the non-normality in the distributions. To statistically test whether there is a difference between the intervention and control groups, the Cochran Armitage test for trend was performed. To statistically test for significance between the FCG and TUG groups for DIH, the numerical scale was retained and the Kruskal–Wallis test was used.

Finally, the differences for the two main outcomes, DIH and number of admissions pre- minus post- intervention were obtained. The mean and standard deviation for the differences were obtained and the change from pre- to post- were analysed using a *t* test (the distribution of the differences was normal). A power analysis indicated that the study was underpowered and results need to be interpreted accordingly. A sensitivity analysis performed to account for the effect of the missing data did not influence the significance of reported DIH or readmissions.

## Results

367 patients were assessed for eligibility to participate in the study, 267 did not meet the inclusion criteria. Five patients (n = 5) declined to participate, eighteen (n = 18) lived outside the designated follow-up area, eight patients (n = 8) did not have reliable contact numbers, twenty-nine (n = 29) had significant medical conditions and two hundred-and-seven patients (n = 207) had alternative diagnoses excluding them from the study. Of the hundred (n = 100) patients who signed informed consent, 49 (n = 49) were allocated to the FCG and 51 (n = 51) to the TUG. At 12-month follow-up, twelve (n = 12) participants in the TUG group could not be located for follow-up. Six (n = 6) participants in the FCG group did not complete the study. Only two participants (n = 2) in the FCG could not be located at 12-month follow up. Of the remaining four that were not included in the final analysis, one had died, one was in prison, one had relocated to another province and the fourth dropped out voluntarily after being included in a medication trial. Data was analysed for 43 (n = 43) participants in the FCG and 39 (n = 39) participants in the TUG.

There were no significant differences in the demographics of the two groups. As expected in this particular catchment area of the Western Cape Metro, the majority (79%) had mixed ethnicity, 77% of participants were single, 88% unemployed and 61% male. Most participants (92%) lived with their families and received disability grants (61%). The incidence of illicit substance use was high in both groups, with 63% of participants (FCG + TGU) admitting to use of illicit substances, 40% indicating that cannabis was their current drug of choice and 14% indicating that methamphetamine was their current drug of choice. The choice of drugs between the FCG and TUG were significantly different (p < 0.001) and 20% of participants in the FCG indicated that methamphetamine was their current drug of choice, compared to 8% in the TUG. There were no other significant differences between the two groups in terms of substance use patterns, but it is important to note that 44% of participants in both groups admitted to frequent use of methamphetamine (weekly + daily). Up to 59% of participants reported frequent use of cannabis.

### 12-Month Pre-inclusion

There were no significant differences in admissions between the two groups at the initial visit (p = 0.48) and there were no significant differences between the groups in terms of the CGI (p = 0.64). There was a marginally significant difference in the DIH for the FCG (mean = 106) compared to the TUG (mean = 85) at the initial visit.

### 12-Month Post-inclusion

There were no differences in readmissions (p = 0.44) and DIH (p = 0.25) at 12-month follow-up. More than a third (34%) of participants in both groups had readmissions over the 12-month period, 12% of participants had more than one admission during this period. Participants in the FCG appeared to be more likely to have more than one readmission (18%) compared to the TUG participants (7%).

According to the CGI, it seemed that more of the TUG participants were severely ill at 12-month follow-up (23% compared to 11% of the FCG participants) and fewer were not ill (21% compared to 35% of the FCG participants; p < 0.05). In terms of how the intervention was experienced, it is interesting to note that the majority of participants did not find the intervention intrusive (72%), 81% felt that the intervention was helpful most or all of the time, yet more than a third of participants did not feel that the intervention helped them to understand their illness better. Staff members reported that only 53.49% of participants engaged well with the intervention and 67.44% of participants were difficult to reach some or all of the time.

We also looked at the difference between the days in hospital (pre-inclusion) and days in hospital (post-inclusion). From this, it is clear that there was a significant drop in the number of days in hospital from pre- to post-intervention (mean difference = 73, SD = 68, *t* test = 10, p < 0.0001). However, the drop in length of stay was similar for both groups (FCG: mean = 78, SD = 69; TUG: mean = 68, SD = 65, *t* test = 0.75, p = 0.455).

## Discussion

Our results indicate that telephone-based facilitation of standard care does not appear to be effective in reducing readmissions and DIH in our setting. This is an interesting finding, considering that similar interventions have been proven to be effective in other settings (Vigod et al. 2013; Nurjannah et al. 2014). Also, previous studies in this setting have successfully demonstrated reduction in DIH in high frequency users (HFUs) when more comprehensive, assertive approaches are used (Botha et al. 2014).

This study was performed in an inpatient system which is under tremendous pressure and where a crisis discharge policy is in place to mediate bed availability. This means that patients who are not well yet, are discharged to make room for patients who are more ill and pose a higher risk to themselves and members of the community. In one local study, patients who had been crisis discharges were found to be more likely to be readmitted (Niehaus et al. 2008). Due to the high turn-over in inpatient wards, patients rarely remain in the ward long enough to receive meaningful psychosocial interventions and such interventions are often difficult to access once discharged. Patients who have not been optimally stabilized upon discharge, may be more likely to become non-compliant soon after discharge, are less likely to engage with outpatient services and more likely to start using substances again (Vigod et al. 2013; Lazarus 2005; Steffen et al. 2009; Nurjannah et al. 2014). It is likely that patients in the FCG were still too ill to benefit from the marginal support this intervention offered. Our findings with regards to participants’ understanding of their illness are interesting, considering other authors reported that lower levels of understanding of case management may lead to early readmission (Sledge et al. 2008).

Although there were no significant differences reported in DIH between the two groups, these findings reflect the importance of including this measure, since there were wide ranges in reported lengths of stay. Our findings showed that participants in the FCG were more likely to have multiple readmissions. The higher incidence of multiple readmissions in the FCG may be a manifestation of the facilitation of care provided by CF’s, who would be able to streamline admissions and intervene early on during relapse. Interestingly, CF’s frequently reported feeling more overwhelmed in supporting FCG participants, compared to the ACT patients that constituted their regular caseload. They accounted this to the fact that home visits allowed for a valuable patient/carer contact during which many crises could be averted. The CF’s often reported that telephone-based contact appeared to be ineffectual in containing carers and engaging with participants. The only recourse in case of crisis was to facilitate an urgent appointment with the CMHN, which often resulted in a readmission due to a lack of other containment options.

The prevalence of substance use in both FCG and TUG group was significant. The past 10 years have seen a sharp rise in methamphetamine abuse in the Western Cape to the extent of being considered a health crisis in the Province (Bateman 2006). A local study published in 2013, reported on the demographic profile of methamphetamine users in psychiatric inpatient units in the Western Cape Province. In 2002, only 0.2% of inpatients reported methamphetamine as their preferred substance. This number increased to 19.3% in 2004 and the 2013 study found that 59% of patients in psychiatric inpatient units reported methamphetamine as their primary substance (Plüddemann et al. 2008). The prevalence of cannabis use also remains quite high. Unpublished data from a study performed in the same unit in 2007, revealed that 73.2% of patients with chronic mental illness reported cannabis as their drug of choice, whereas only 11.3% reported methamphetamine as their drug of choice (Botha et al. 2010). Though the prevalence of substance use was not the focus of this study, our findings highlight the sharp rise in especially methamphetamine use in the province. It is likely that the incidence of substance use, especially methamphetamine use, had an impact on the effect of the intervention in this study.

Although the findings of this study need to be interpreted in the context of a small sample size, the results indicate that telephone-based facilitation of standard care may not be an effective post-discharge intervention in our particular setting. It is likely that the outcomes reflect the fact that the telephone-based intervention was simply not comprehensive enough to support this population of patients effectively in this specific setting. The combination of premature discharges, high rates of substance use and an overburdened outpatient service, make it difficult to facilitate care effectively. This study also demonstrates the impact unique substance use trends (particularly methamphetamine in this setting) may have on inpatient services and consequently, post-discharge services. At face value, it may seem that we are reporting on an unsuccessful intervention. However, this is an important finding, since telephone-based facilitation may offer a more affordable alternative to comprehensive post-discharge care, but does not seem to be effective in reducing inpatient usage in our setting. We are therefore still in search of an effective and affordable intervention that is practical an under-resourced setting and accessible to a wide range of patients.

## Conclusion

Telephone-based facilitation of existing standard care services in this setting did not have any impact on readmission rates or DIH for mental health care users. There is still a need for further exploration of affordable and practical post-discharge services that impact on inpatient service use. Our study also identifies the need for services that incorporate a unique approach to support the distinct population of substance using mental health service users.

## Limitations

The authors acknowledge some limitations to the study. Firstly, the small sample size implies that any conclusions drawn from the study should be interpreted with caution. The study was piloted as a single-site study in a high-pressure area and the intervention itself was provided by members of the ACT Team. As such, inclusion rates were limited by existing case-loads and service requirements, which accounts for the small sample size. A power analysis confirmed that the study is under-powered and results should be interpreted as such. For the study to have been adequately powered, we would have required 220 participants, which was not feasible in the context of the service pressure. Some of the exclusion criteria (such as not having access to a telephone) may have biased the sample characteristics and access to services. Also, data collection in a pre-discharge population could be biased as service users may be reluctant to acknowledge some aspects of history that they feel could impact on the discharge decision. Although a sensitivity analysis indicated that the effect of the missing data did not influence the significance of the reported results, it is possible that the missing post-inclusion data was not random and that this could have influenced the results. This telephone-based intervention for patients with severe mental illness should be duplicated in other studies and other scenarios before accepting the (negative) outcomes of the current study.
